# The Effect of Dalbavancin in Moderate to Severe Hidradenitis Suppurativa

**DOI:** 10.3390/antibiotics11111573

**Published:** 2022-11-08

**Authors:** Elisa Molinelli, Claudia Sapigni, Giovanni Marco D’Agostino, Valerio Brisigotti, Giulio Rizzetto, Ivan Bobyr, Oscar Cirioni, Andrea Giacometti, Lucia Brescini, Sara Mazzanti, Annamaria Offidani, Oriana Simonetti

**Affiliations:** 1 Dermatological Unit, Department of Clinical and Molecular Sciences, Polytechnic Marche University, 60121 Ancona, Italy; 2 Clinic of Infectious Diseases, Department of Biochemical Sciences and Public Health, Polytechnic University of the Marche Region, 60121 Ancona, Italy

**Keywords:** hidradenitis suppurativa, dalbavancin, antibiotics, treatment, biologic agents

## Abstract

Hidradenitis suppurativa (HS) is a chronic inflammatory skin disease characterized by painful nodules, abscesses, and fistulas, localized to the areas of the folds where apocrine glands are present: the armpits, groin, inframammary region, and genital or perineal region. The management is still challenging, and it includes mainly systemic antibiotics, immunosuppressors, and biologic agents. Antibiotics are frequently used in the management of HS for their anti-inflammatory, immunomodulatory, and antimicrobial properties, but no data have been reported regarding the use of dalbavancin in HS. The aim of our practice was to evaluate efficacy, flare, and disease-free survival after dalbavancin therapy in a selected population with HS. We report the experience of the Ancona Dermatology Clinic in treating HS flare-ups with dalbavancin and its rationale for use. Our observation shows that the use of dalbavancin is an effective and well-tolerated treatment for the management of Hurley stage II-III HS; currently, dalbavancin should be considered as a supportive therapy for selected patients.

## 1. Introduction

Hidradenitis suppurativa (HS) is a chronic inflammatory skin disease involving the intertriginous areas where apocrine glands are present and is characterized by a progression from nodules to inflamed, painful, deep-seated abscesses and draining fistulas with persistent suppuration and, ultimately, irreversible fibrotic scars [1].

The debilitating consequences of a failure to diagnose or improper therapeutic management lead to severe pain and irreversible skin manifestations (e.g., fistulas, disfiguring scars). HS was found to significantly impair patients’ quality of life to a greater extent than other skin conditions. Adequate, early recognition and treatment are critical for a favorable prognosis.

The pathogenesis of HS is still debated even though significant progress has been made in understanding it in recent years: (i) dysregulated gene pathways in typical HS lesions were mapped, (ii) genetic and microbiome contributions to disease were investigated [2].

Although HS is not primarily caused by bacterial infection, it is possible to isolate different bacterial species from the exudate of HS lesions. Studying the bacteriological contribution in the pathogenesis of HS lesions, a polymicrobial microflora characterized by *coagulase-negative staphylococcal species* and *mixed anaerobic bacteria*, with *Staphylococcus aureus* and *streptococcal species*, was identified [3].

Bacterial dysbiosis, which characterizes HS lesions, is an important component of the vicious cycle of inflammation in several dermatologic diseases, and probably acts by presenting inflammatory signals and molecular targets to the immune system [3].

As the exact pathogenesis of HS is still unclear, treatment is still challenging and not yet standardized, but antibiotics represent the first-line treatment in HS mainly for their anti-inflammatory properties alongside antimicrobial and immunomodulatory effects [4].

According to the 2019 North American guidelines, tetracyclines are recommended in mild to moderate HS, for a 12-week period or as long-term maintenance, when appropriate. Clindamycin and rifampin, in combination, are effective as a second-line treatment for mild to moderate disease or as a first-line treatment or adjunct treatment in severe disease [4].

Adalimumab, the only biological drug currently indicated for the treatment of HS, at the approved HS dosing, is recommended to improve disease severity and quality of life in patients with moderate to severe HS [4].

## 2. Case Description

Although the role of antibiotics (ATBs) in the management of HS is still debated, we report the experience of the Dermatological Clinic of Ancona in the treatment of HS flares with dalbavancin and its rationale for use. We treated eight patients with moderate to severe HS with dalbavancin. All members of the population were examined clinically and ultrasonographically at all sites affected by the disease; all met the clinical criteria for the diagnosis of HS according to international HS guidelines, with a Hurley score between I and III [5].

The patients ranged in age from 17 to 59 years. The sites involved were the groin in all patients, the armpits in five patients, the genital region in two patients, the mammary region in two patients, the perineal and perianal sites in one patient, the buttocks site in two patients, and the pubis site in two patients. The family history was positive for HS in two patients. Four patients had a positive history for acne vulgaris and two of these also for pilonidal cysts. All patients had previously been treated with topical and systemic drugs including tetracyclines (300 mg of lymecycline or 100 mg of minocycline); two patients had received clindamycin and rifampin in combination, three patients had received 25 mg of acitretin, and six patients (three previous and three current) had received adalimumab (Table 1). 

Although the patients were being treated with adequate therapies, frequent exacerbations were present, with a major impact on quality of life. 

At our first observation (T0), patients presented an exacerbation phase with a Hurley score of II/III, average Hidradenitis Suppurativa Severity Score System (IHS4) score of 19, average pain Visual Analogue Scale (VAS) score of eight, and average Dermatological Quality of Life Index (DLQI) score of 26. Patients were treated with 100 mg of dalbavancin IV as a slow infusion and re-evaluated at 12 (T12) and 24 weeks (T24) (Table 2).

## 3. Results

The eight patients were re-evaluated at 12 and 24 weeks. In addition to baseline scores, disease-free survival (DFS) and Hidradenitis clinical response (HiSCR) were recorded. DFS is defined as the length of time the patient has no disease; HiSCR is a clinical endpoint useful for evaluating therapeutic outcomes in patients with HS. HiSCR is defined as a reduction of at least 50% in total nodule count with no increase in abscess count and no increase in drainage fistula count from baseline [6].

Significant disease improvement was achieved at 12 weeks (T12) with average values of 7 for IHS4, 2 for pain VAS, and 8 for DLQI, and HiSCR was satisfied in six out of eight patients compared to baseline (T0) (Table 3).

The following mean values were recorded at our last clinical evaluation at 24 weeks: 10 for IHS4, 3 for pain VAS, 10 for DLQI, and 15-weeks for DFS on average; the HiSCR was satisfied in three out of eight patients compared to baseline (T0) (Table 4 and Table 5).

## 4. Discussion

Antibiotic therapy with dalbavancin was performed with the support of several considerations. HS is a chronic inflammatory disease, and the bacteria role is still debated; it is not primarily caused by bacterial infection, but many different bacterial species can often be isolated from HS lesions. In HS lesions, a mixed microbial flora can be isolated, different from that identified in healthy skin, with a predominance of *coagulase negative staphylococcal species*, *mixed anaerobic bacteria*, *Staphylococcus aureus*, and *streptococcal species*. Commensal and pathogenic bacteria colonizing lesions may play a role either as an inflammatory trigger, presenting inflammatory signals and molecular targets to the immune system, or they may act as actual infectious agents. In the treatment of HS, ATBs have demonstrated effectiveness. ATBs administration exerts its therapeutic effect through anti-infective activity, which kills or inhibits bacterial proliferation by disrupting key pathways of bacterial metabolism or replication, and through anti-inflammatory mechanisms, inhibiting lymphocyte proliferation, T-cell activity, neutrophil activity, and suppressing tumor necrosis factor-α secretion [3,7].When ATBs are administered primarily for antimicrobial reasons, optimal clinical management should consider the use of ATBs targeted to bacterial strains isolated after culture examination. If, however, the therapeutic goal is primarily anti-inflammatory, it must consider that the administration of ATBs exposes the patient’s entire commensal flora to ATBs, potentially inducing antibiotic resistance [3,7].

Considering the emerging antimicrobial resistance and the consequent reduced efficacy of the antibiotic therapies used in the treatment of HS, ATBs should be administered only when clinically indispensable and whenever HS purulent material is available [8].

Furthermore, keeping in mind that the use of broad-spectrum antibiotics is still of primary importance in major infectious diseases, such as rifampin therapy in the treatment of active tuberculosis, it would be reasonable, based on these assessments, to use alternative, targeted antibiotics in the treatment of HS [8]. Therefore, it was deemed appropriate to perform a deep swab on purulent material of draining abscesses or fistulas and/or biopsy sampling of inflammatory lesions; to obtain a culture test and consequently perform a targeted therapy. Culture tests performed on the HS lesions of our eight patients were positive for the presence of bacteria such as *Staphylococcus aureus*, *Streptococcus agalactiae*, *Enterococcus fecalis*, and *Proteus*.

Considering the prevalence of Gram-positive bacteria, we decided to treat patients with a single intravenous dose of 1500 mg of dalbavancin as a slow infusion in the hospital setting.

Currently, bactericidal lipoglycopeptide and glycopeptide antibiotics, such as daptomycin, teicoplanin, and dalbavancin, have a significant role in therapy against Gram-positive bacterial infections and are usually recommended for the treatment of complicated skin, soft tissue, and bloodstream infections caused by *S. aureus* [9, 10, 11].

Dalbavancin is a semisynthetic molecule derived from a glycopeptide, which is approved exclusively for the treatment of acute bacterial skin infections sustained by Gram-positive bacteria, including meticillin-resistant and methicillin-sensible *Staphylococcus aureus*, *Streptococcus pyogenes*, *Streptococcus agalactiae*, and *Enterococcus faecalis* [12]. These bacteria have been strongly associated with the HS microbiome, which is also composed to a lesser extent by Gram-negative and anaerobic bacteria [12]. Dalbavancin has an excellent safety profile. The most frequent adverse events are infusion-related effects (pruritus, flushing), gastrointestinal disorders (nausea, diarrhea), and headache [13]. No data on the anti-inflammatory role of dalbavancin are available.

In 2020, Simonetti et al. demonstrated that dalbavancin, in addition to having an antibacterial activity, also plays a pivotal role in stimulating a faster and qualitatively better tissue repair process. Reducing MMP-1 and MMP-9 and increasing expressions of EGFR and VEGF, dalbavancin induced the proliferation and migration of keratinocytes in the epidermis and neo angiogenesis in a mouse model wound healing [14].

Biologic agents, specifically anti-TNF alpha, have revolutionized the therapeutic armamentarium of several immune-mediated diseases such as psoriasis, atopic dermatitis, and HS [15].

As HS is an immune-mediated inflammatory disease, and we can argue that biological agents are now the gold standard. In fact, in the literature, a growing number of biological and small molecule drugs are proposed for the management of the disease. In light of recent antibiotic resistance considerations, this should be considered in the process of updating guidelines for the treatment of HS.

It will, therefore be, necessary to investigate the role of dalbavancin in HS and to clarify its anti-inflammatory and immunomodulatory potential.

Currently, dalbavancin should be considered as a supportive therapy in selected patients in order to: (i) control disease flare-ups; (ii) have bridging therapy available with surgical therapy; (iii) support biologic therapy with adalimumab, e.g., in case of a loss of efficacy, in case of flare-ups during therapy, or in case of contraindication to the biologic drug; (iv) have specific therapy for skin infections and avoid broad-spectrum antibiotic therapies while trying to reduce the emergence of resistance.

Prospective and randomized controlled studies are needed to verify the efficacy of dalbavancin in HS and to further elucidate the role of this antibiotic in the disease. 

## Figures and Tables

**Table 1:** Demographic and clinical characteristics of HS patients.

Patients	Gender	Age	Age of Onset	Affected Areas	Family History	Comorbidities	Medical History	BMI	Smoke	Previous Treatment	Concomitant Treatment
1	F	38	34	Groin Armpits	Neg	Acne	SLE PCOS Hashimoto thyroiditis	24	Pos	Lymecycline Minocycline	/
2	M	51	12	Groin Perineal Buttocks	Pos	Acne Pilonidal cyst	Metabolic syndrome	33	Pos	Lymecycline Minocycline Acitretin Rifampicin +clindamycin	Adalimumab
3	M	54	40	Groin Armpits Genital Buttocks	Neg	Acne Pilonidal cyst	Previous latent TBC	28	Neg	Lymecycline Minocycline Acitretin Rifampicina +clindamycin Adalimumab	/
4	F	19	14	Groin Armpits Mammary	Pos	Neg	Neg	26	Neg	Lymecycline	Adalimumab
5	F	37	13	Groin Armpits Mammary	Neg	Acne	Neg	26	Pos	Lymecycline Isotretinoin Adalimumab	/
6	F	59	25	Groin Genital Pubis	Neg	Neg	Obesity DMT2	31	Pos	Adalimumab Minocycline	/
7	F	59	22	Groin Pubis Abdomen	Neg	Neg	Obesity	30	Neg	Minocycline Acitretin	/
8	F	17	13	Groin Armpits	Neg	Neg	Obesity	35	Neg	Minocycline	Adalimumab

BMI: body mass index; SLE: systemic lupus erythematosus; PCOS: polycystic ovary syndrome; TBC: tuberculosis; DMT2: type 2 diabetes mellitus.

**Table 2:** Clinical assessment at baseline (T0).

Patients	Hurley	IHS4	DLQI	Pain VAS	Total Lesions	Nodules	Draining Fistulas	Abscesses
1	II	12	21	7	5	2	2	1
2	III	20	27	7	9	4	3	2
3	III	31	24	8	12	5	6	1
4	III	17	28	9	7	3	3	1
5	III	23	30	10	10	5	4	1
6	II	15	25	9	7	3	2	2
7	III	17	24	8	7	3	3	1
8	II	16	28	7	9	6	2	1
Average values	/	19	26	8	/	/	/	/

IHS4: Hidradenitis Suppurativa Severity Score System; DLQI: Dermatological Quality of Life Index; VAS: Visual Analogue Scale.

**Table 3:** Clinical assessment at week 12 (T12).

Patients	Hurley	IHS4	DLQI	Pain VAS	Total Lesions	Nodules	Draining Fistulas	Abscesses	HiSCR
1	II	1	6	0	1	1	0	0	Y Y Y
2	III	7	10	0	4	3	1	0	Y Y Y
3	III	16	10	0	7	4	3	0	Y Y N
4	III	2	7	1	2	2	0	0	Y Y Y
5	III	16	12	3	7	4	3	0	Y Y N
6	II	5	5	2	2	1	1	0	Y Y Y
7	III	5	5	4	2	1	1	0	Y Y Y
8	II	7	7	3	4	3	1	0	Y Y Y
Average values	/	7	8	2	/	/	/	/	/

IHS4: Hidradenitis Suppurativa Severity Score System; DLQI: Dermatological Quality of Life Index; VAS: Visual Analogue Scale; HiSCR: Hidradenitis Suppurativa Clinical Response.

**Table 4:** Clinical assessment at week 24 (T24).

Patients	Hurley	IHS4	DLQI	Pain VAS	Total Lesions	Nodules	Draining Fistulas	Abscesses	Flare/24 Weeks	DFS	HiSCR
1	II	3	7	1	3	1	0	2	1	22	Y N N
2	III	10	12	3	5	2	1	2	2	15	Y Y N
3	III	20	18	6	7	2	4	1	2	17	Y Y Y
4	III	6	7	1	3	2	1	0	1	19	Y Y Y
5	III	16	12	5	8	4	3	1	4	4	Y Y N
6	II	6	4	0	3	2	1	0	2	16	Y Y Y
7	III	9	6	4	5	3	1	1	1	18	Y Y N
8	II	10	12	5	6	4	1	1	3	7	Y Y N
Average values	/	10	10	3	/	/	/	/	/	15	

IHS4: Hidradenitis Suppurativa Severity Score System; DLQI: Dermatological Quality of Life Index; VAS: Visual Analogue Scale; DFS: disease-free survival; HiSCR: Hidradenitis Suppurativa Clinical Response.

**Table 5:** Clinical assessment at baseline, week 12 and week 24.

	Baseline			Week 12			Week 24		
Patients	IHS4	DLQI	Pain VAS	IHS4	DLQI	Pain VAS	IHS4	DLQI	Pain VAS
1	12	21	7	1	6	0	3	7	1
2	20	27	7	7	10	0	10	12	3
3	31	24	8	16	10	0	20	18	6
4	17	28	9	2	7	1	6	7	1
5	23	30	10	16	12	3	16	12	5
6	15	25	9	5	5	2	6	4	0
7	17	24	8	5	5	4	9	6	4
8	16	28	7	7	7	3	10	12	5
Average values	19	26	8	7	8	2	10	10	3

IHS4: Hidradenitis Suppurativa Severity Score System; DLQI: Dermatological Quality of Life Index; VAS: Visual Analogue Scale.

## Data Availability

The data presented in this study are available in supplementary materials here (Table 1, Table 2, Table 3 and Table 4).
